# *MLH1* V384D polymorphism associates with poor response to EGFR tyrosine kinase inhibitors in patients with *EGFR* L858R-positive lung adenocarcinoma

**DOI:** 10.18632/oncotarget.3511

**Published:** 2015-03-10

**Authors:** Chao-Hua Chiu, Hsiang-Ling Ho, Howard Doong, Yi-Chen Yeh, Mei-Yu Chen, Teh-Ying Chou, Chun-Ming Tsai

**Affiliations:** ^1^ Institute of Clinical Medicine, National Yang-Ming University, Taipei, Taiwan; ^2^ Department of Medicine, National Yang-Ming University, Taipei, Taiwan; ^3^ Institute of Biochemistry and Molecular Biology, National Yang-Ming University, Taipei, Taiwan; ^4^ Division of Thoracic Oncology, Department of Chest Medicine, Taipei Veterans General Hospital, Taipei, Taiwan; ^5^ Division of Molecular Pathology, Department of Pathology and Laboratory Medicine, Taipei Veterans General Hospital, Taipei, Taiwan; ^6^ Taipei VGH-Lihpao Laboratory of Cancer Genomic Medicine, Lihpao Life Science Corporation, Taipei, Taiwan

**Keywords:** MLH1, EGFR, Lung adenocarcinoma, Tyrosine kinase inhibitor, Resistance

## Abstract

A significant fraction of patients with lung adenocarcinomas harboring activating epidermal growth factor receptor (EGFR) mutations do not experience clinical benefits from EGFR tyrosine kinase inhibitor (TKI) therapy. Using next-generation sequencing, we screened 739 mutation hotspots in 46 cancer-related genes in *EGFR* L858R-mutant lung adenocarcinomas from 29 patients who received EGFR-TKI therapy; 13 had short (< 3 months) and 16 had long (> 1 year) progression-free survival (PFS). We discovered *MLH1* V384D as a genetic variant enriched in the group of patients with short PFS. Next, we investigated this genetic variation in 158 lung adenocarcinomas with the *EGFR* L858R mutation and found 14 (8.9%) patients had *MLH1* V384D; available blood or non-tumor tissues from patients were also tested positive for *MLH1* V384D. Patients with *MLH1* V384D had a significantly shorter median PFS than those without (5.1 vs. 10.6 months; P= 0.001). Multivariate analysis showed that *MLH1* V384D polymorphism was an independent predictor for a reduced PFS time (hazard ratio, 3.5; 95% confidence interval, 1.7 to 7.2; P= 0.001). In conclusion, *MLH1* V384D polymorphism is associated with primary resistance to EGFR-TKIs in patients with *EGFR* L858R-positive lung adenocarcinoma and may potentially be a novel biomarker to guide treatment decisions.

## INTRODUCTION

Lung cancer has high incidence rates worldwide, and its 5-year survival is dismal as most cases are diagnosed at late stages. Chemotherapy, although with limited efficacy, used to be the main treatment option for patients with advanced lung cancer [1]. In 2004, somatic mutations were reported to exist in the tyrosine kinase domain of epidermal growth factor receptor (EGFR) in tumors of a subset of patients with non-small cell lung cancer (NSCLC) who responded dramatically to EGFR tyrosine kinase inhibitors (TKIs) [2, 3]. This discovery has opened a new era of targeted therapy for NSCLC. Nowadays, EGFR-TKIs are used as the standard first-line therapy for patients with advanced lung adenocarcinoma harboring activating *EGFR* mutations [4, 5], and they remarkably improve the survival and quality of life in patients with these driver mutations [6].

Drug resistance is a major obstacle in targeted cancer therapy, and understanding the mechanisms of resistance is pivotal for developing more effective treatment strategies. Around 70% of patients with lung adenocarcinoma that has activating *EGFR* mutations (mostly a small in-frame deletion in exon 19 and a substitution mutation L858R) display objective clinical response to EGFR-TKI treatment [7-11]. However, despite the initial disease control, tumor relapse is inevitably observed after a median of 9-14 months, indicating the development of acquired resistance to EGFR-TKIs in these patients [7-9, 11]. Studies have identified different mechanisms of acquired EGFR-TKI resistance, including a second-site *EGFR* T790M mutation [12, 13], *MET* amplification [14, 15], *PIK3CA* mutations [16], FGFR1 activation [17], epithelial-to-mesenchymal transitions and conversion to small cell carcinoma [16, 18-19]. On the other hand, ~30% patients with TKI-sensitive *EGFR* mutations fail to demonstrate objective tumor regression on initial EGFR-TKI therapy and are defined as having primary or intrinsic resistance [7,9, 11]. Some co-existing genetic variations have been implicated in the mechanism of TKI insensitivity in *EGFR*-mutant patients, including de novo presence of *EGFR* T790M or *MET* amplification [20, 21], *KRAS* mutations [22], loss of *PTEN* [23], and a germline deletion polymorphism of *BIM* [24]. However, the majority of resistant cases cannot be explained by these variations and the mechanistic basis for intrinsic EGFR-TKI resistance in patients supposed to be responsive is still largely unknown.

In this study, we hypothesized that specific genetic alterations may underlie the primary resistance to *EGFR*-TKIs in lung adenocarcinoma harboring activating *EGFR* mutations. Towards uncovering such genetic determinants of treatment resistance, we performed next-generation sequencing (NGS)-based mutation profiling of lung adenocarcinomas with the *EGFR* L858R mutation from patients who received EGFR-TKI therapy, and searched for genetic variants/mutations that could differentiate patients displaying primary resistance to EGFR-TKIs from those having a durable response.

## RESULTS

### Forty-six-gene mutation profiles of *EGFR* L858R-positive lung adenocarcinomas

**Figure 1:**
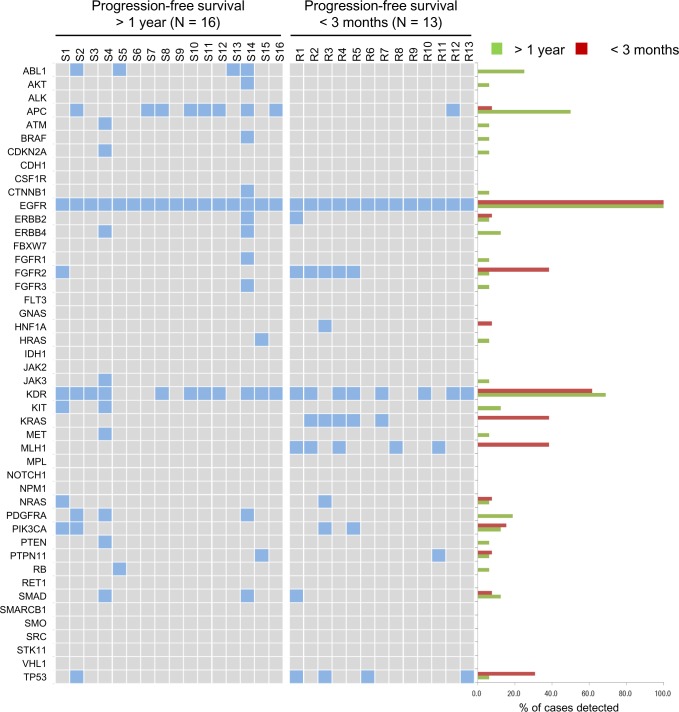
Genetic variations of *EGFR* L858R lung adenocarcinomas in 46 cancer-related genes The mutation profiles in hotspot regions of 46 cancer-related genes in individual *EGFR* L858R tumors are shown on the left in 2 groups, according to the progression-free survival of patients. A positive result for mutation is indicated by a filled box. The percentage of mutation identified in each group for each gene is shown on the right.

NGS was used to interrogate mutations within hotspot regions of 46 cancer-related genes in lung adenocarcinoma samples from 13 and 16 EGFR-TKI-treated patients who had short (< 3 months) and long (> 1 year) PFS, respectively. Differential mutation patterns were revealed in these two groups (Fig. 1 and more details in the Supplementary Appendix). All 29 tumors were confirmed to harbor the activating *EGFR* L858R mutation without the simultaneous presence of the T790M allele that predicts EGFR-TKI resistance. Among the 46 genes, *KDR* (which encodes for vascular endothelial growth factor receptor 2) was the most commonly mutated gene coexisting with *EGFR* L858R, regardless of the patient's treatment response. Mutation rates of *ABL1*, *APC*, and *PDGFRA* were disproportionately high in the patient with long PFS. In contrast, mutations in *FGFR2* (K368E), *KRAS* (G12D), *MLH1* (V384D), and *TP53* occurred more often in patients with short PFS. Derepression of *FGFR2* expression has been implicated in the mechanism for rapidly acquired EGFR-TKI resistance in NSCLC cells [12]. *KRAS* G12C is linked to poor outcomes of EGFR-TKI therapy in NSCLC patients [13]. A study shows higher p53 mutation rates in advanced-stage than in early-stage lung cancers and suggests that the concurrent occurrence of p53 mutations with EGFR mutations may foster the development of therapeutic resistance [25]. However, the DNA mismatch repair gene *MLH1* has not been associated with EGFR-TKI resistance yet.

### *MLH1* V384D in patients with primary lung adenocarcinoma

**Figure 2:**
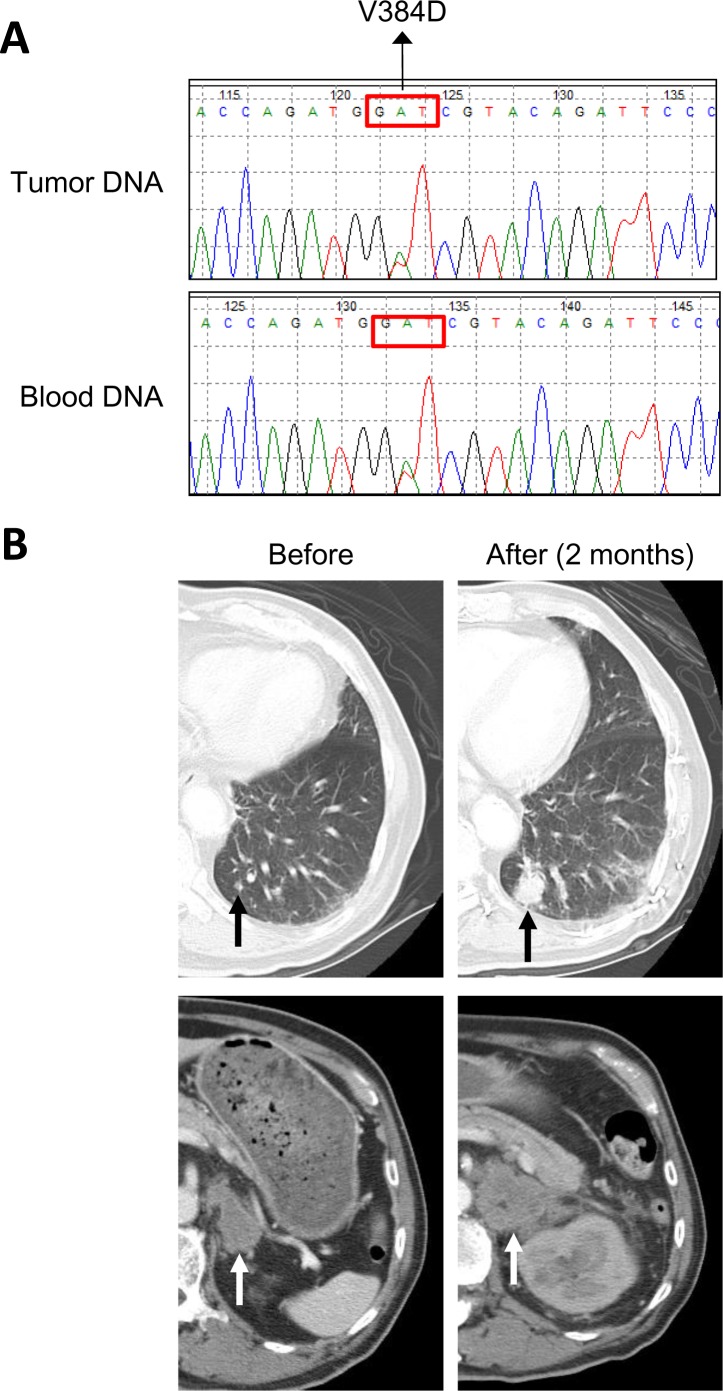
*MLH1* V384D polymorphism in a lung adenocarcinoma patient with EGFR-TKI resistance Panel A shows the results of Sanger sequencing of the *MLH1* gene in both tumor and blood DNA specimens from a representative patient with a heterozygous T→A substitution at nucleotide 1151, which results in a Val384Asp substitution in MLH1. Panel B shows the chest CT scans of a representative patient with the *MLH1* V384D polymorphism, demonstrating the persistent growth of metastatic lesions in lung (black arrows) and adrenal gland (white arrows) during erlotinib treatment.

We examined the mutation status of *MLH1* in a larger set of *EGFR* L858R-positive lung adenocarcinomas. A total of 158 tumors were subjected to *MLH1* mutation analysis by direct sequencing of PCR products. Fourteen of the 158 tumors (8.9%) had a heterozygous T→A change at nucleotide 1151 (Fig. 2A) which results in the same V384D substitution in MLH1 as discovered in NGS screening. We were able to analyze genomic DNA from blood specimens of 4 patients and non-tumor tissue specimens from 1 patient, and all of these samples were tested positive for *MLH1* V384D (as in Fig. 2A). Clinical characteristics of patients with or without *MLH1* V384D were analyzed (Table 1), and no statistically significant demographic differences between the two groups were noted. We also performed sequencing analysis of *MLH1* exon 12 in 51 *EGFR*-wildtype lung adenocarcinomas and found a comparable incidence (4/51, 7.8%) of the *MLH1* V384D allele.

**Table 1:** Patient characteristics (n= 158)

	*MLH1* codon 384		P value
	V/V	V/D	
Total case number	144	14	
Gender			0.577
Male	50	5	
Female	94	9	
Age			0.240
Median	65	60	
(Range)	(38-94)	(43-78)	
Smoking			0.096
Never	111	8	
Ever	33	6	
Stage			0.119
IIIB	5	2	
IV	139	12	
Number of prior chemotherapy			0.661
0	116	12	
1	24	2	
2	4	0	
EGFR mutation			0.756
L858R	141	14	
L858R, complex	3	0	
EGFR-TKI			0.897
Gefitinib	120	12	
Erlotinib	23	2	
Afatinib	1	0	

### Tumor response to EGFR-TKI

**Figure 3:**
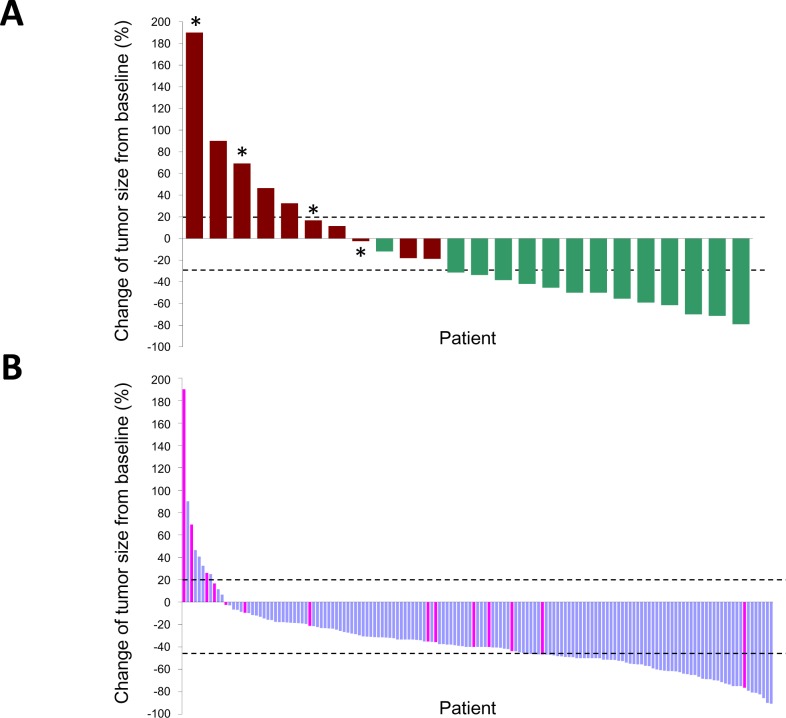
Waterfall plots of the maximum percentage change in tumor size of individual *EGFR* L858R lung adenocarcinomas treated by EGFR-TKIs Tumors are listed in order of increasing extent of response to EGFR-TKIs; only those with measurable sizes before and after EGFR-TKI treatment are shown. The upper (20%) and lower (−30%) dashed lines indicate the thresholds used to define a progressive disease and a partial response, respectively, by the RECIST criteria. Panel A shows individual tumor responses in 24 patients analyzed by NGS. Red bars, PFS < 3 months; green bars, PFS < 1 year; asterisks, positive for *MHL1* V384D. Panel B shows 155 *EGFR* L858R tumors analyzed for *MLH1* status by direct sequencing of PCR products. Pink bars, positive for *MLH1* V384D.

A representative tumor with concurrent *EGFR* L858R and *MLH1* V384D mutations displayed primary resistance to EGFR-TKI treatment (Fig. 2B). We evaluated individual tumor responses to EGFR-TKIs in patients whose tumors were of measurable sizes. Twenty-four of the NGS-screened 29 patients were monitored, and the tumor responses and PFS clustered correspondingly (Fig. 3A); 5 of 10 (50%) patients with short PFS had progressive disease whilst on EGFR-TKI treatment and 13 of 14 (92.9%) patients with long PFS had a partial response to EGFR-TKIs. Among the 4 patients with the *MLH1* V384D allele, 2 had progressive disease and 2 had stable disease; none of the patients with partial response carried the *MLH1* V384D allele.

In the 158 patients examined for *MLH1* mutations by PCR and Sanger sequencing, 155 had measurable tumors and their responses to EGFR-TKIs were monitored (Fig. 3B). The overall response rate was 69.7%; 108, 39 and 8 patients achieved partial response, stable disease and progressive disease, respectively. The response rates for tumors with and without *MLH1* V384D mutation were 50% and 71.6%, respectively (P= 0.088). *MLH1* V384D-positive tumors had a smaller size reduction in response to EGFR-TKI treatment than that in tumors without the allele (median size change −28.2% vs. −40.5%, P= 0.015, Mann-Whitney U test). The *MLH1* V384D allele was over-represented in patients with EGFR-TKI resistance. Only 11 of 155 (7.1%) *EGFR* L858R-positive tumors showed disease progression under EGFR-TKI treatment, and 4 of these 11 (36.4%) had *MLH1* V384D. Among the 144 tumors either showing a partial response or being stable on treatment, only 10 (6.9%) were *MLH1* V384D-positive.

### Survival analysis

**Figure 4:**
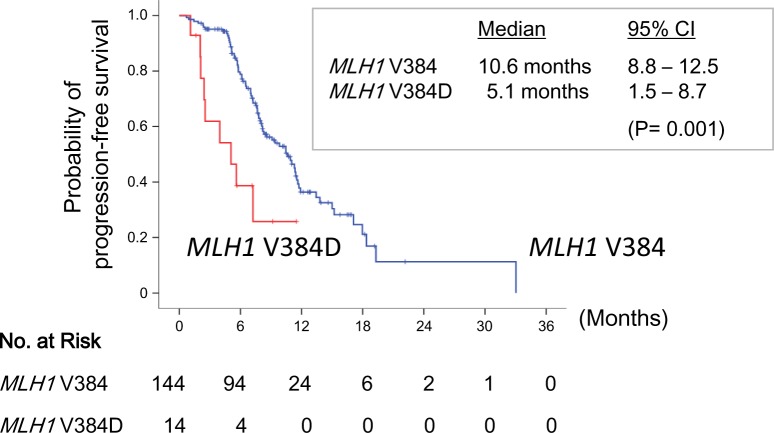
Progression-free survival stratified by *MLH1* V384 status Kaplan-Meier curves of progression-free survival in patients with *EGFR* L858R lung adenocarcinoma who were treated by EGFR-TKIs, according to the presence (red line) or absence (blue line) of the *MLH1* V384D polymorphism.

At the time of analysis, with a median follow-up of 47.4 months, 51 patients remained in use of an EGFR-TKI and 107 patients (67.7%) had experienced PFS. The overall median PFS was 10.5 months (95% CI, 8.1 to 12.8 months). Patients with the *MLH1* V384D mutation had a significant shorter PFS (median, 5.1 months; 95% CI, 1.5 to 8.7 months) than that of those without (median, 10.6 months; 95% CI, 8.8 to 12.5 months) (P= 0.001) (Fig. 4). Gender (male vs. female, P= 0.031) and the number of prior chemotherapy (0 vs. ≥ 1, P= 0.002) were also predictor variables for PFS. In the multivariate analysis using the Cox regression model, only the number of prior treatment (HR= 2.3, 95% CI, 1.4 to 3.8; in favor of none; P= 0.001) and the *MLH1* mutation status (HR= 3.5, 95% CI, 1.7 to 7.2; in favor of no V384D mutation; P= 0.001) were independent predictors for PFS.

## DISCUSSION

Our NGS-based multi-gene mutation profiling of lung adenocarcinomas carrying the activating EGFR mutation L858R has uncovered an *MLH1* V384D allele that is over-represented in a subset of tumors showing primary resistance to EGFR-TKIs. We have validated the disproportionately high occurrence of the *MLH1* V384D allele in patients showing poor response to EGFR-TKI treatment, and demonstrated that *MLH1* V384D is associated with a shorter PFS.

MLH1 is a component of the cellular DNA mismatch repair (MMR) machinery [26]. The MMR system is responsible for the recognition and repair of single-base mismatches and short insertions/deletions that may arise during DNA replication and recombination. Inherited mutations in the MMR system can cause genomic instability and human diseases such as Lynch syndrome; individuals with this syndrome are at high risk of colon cancer and other malignancies. Recognition of DNA damage by the MMR system is instrumental for activation of the apoptosis cascade; therefore, cells with MMR dysfunction may not properly induce apoptosis in response to DNA damage. Indeed, dysfunctional MMR has been implicated in the mechanism of resistance to DNA-damaging chemotherapeutics such as cisplatin [27]. However, to our knowledge, *MLH1* and other MMR genes have never been linked to EGFR-TKI resistance.

Although many *MLH1* variants are known [28, 29], only the V384 variant was identified in this study. *MLH1* V384D was first identified in Chinese colorectal cancer patients and shown to be a common (2.5-2.67% in normal individuals) germline polymorphism in the East-Asian population [29, 30]. The detection of *MLH1* V384D in blood or non-tumor tissue samples of patients with *EGFR* L858R-mutant lung adenocarcinoma also suggests that it is not a somatic mutation but a germline mutation/polymorphism. Similar to the reported 7.7% allele frequency in Chinese colorectal cancer patients [30], we found an incidence of *MLH1* V384D around 7-8% in tumors with wild-type or L858R mutant *EGFR*, suggesting that *MLH1* V384D may not be a mutation secondary to the *EGFR* L858R mutation. Whether the presence of *MLH1* V384D increases the rates of activating *EGFR* mutations requires further investigation to determine.

The exact mechanism of how the MLH1 V384D variant influences EGFR-TKI treatment response on *EGFR* L858R-mutant tumors is not clear at present. A few possible scenarios are hereby presented: i) The MLH1 V384D variant is, at least partially, impaired in its protein function, displaying decreased efficiency in the interaction with its partner protein PMS2 and showing reduced MMR activity *in vitro* [28, 29]. As MMR contributes to genome stability, cancer cells with *MLH1* V384D may hence be prone to accumulating mutations during cancer progression. This mutator phenotype could facilitate the acquisition of additional driver mutations in alternative proliferation or survival pathways, and enable cancer cells to become resistant to EGFR-targeting therapies. ii) Activated EGFR can translocate from cell membrane into the nucleus to execute several important functions, including DNA repair [31]. EGFR-TKI therapy inhibits the kinase activity and interferes with nuclear translocation of EGFR, which may possibly increase DNA damage. Because MLH1 is responsible for recognizing DNA damage and activating the apoptotic pathway, cells expressing the functionally impaired MLH1 V384D variant may not enter apoptosis efficiently and thus still display unchecked proliferation under EGFR-TKI treatment. iii) Besides mechanisms attributable to MMR dysfunction, we cannot exclude the possibility that MLH1 may have MMR-independent functions involved in EGFR signaling or other proliferation or survival pathways. Further investigation is warranted to elucidate the principal mechanism underlying the MLH1 V384D-associated EGFR-TKI resistance.

This study has important clinical implications. Instead of discovering additional “driver” mutations, we have identified a germline polymorphism associating with primary resistance to EGFR-TKIs. The first implication of this study is that: not only pathway-activating mutations in tumors but also germline genetic variations in individuals should be considered for molecular testing in the era of “personalized therapy”. In line with this notion, both tumor and blood samples should be archived to facilitate future translational studies. Secondly, our findings suggest that the combination of EGFR-TKIs with other anti-cancer therapeutics may be a rational treatment strategy for patients with lung adenocarcinomas concurrently harboring a somatic *EGFR* L858R mutation in the tumor and a germline *MLH1* V384D polymorphism. In fact, combination therapy has been proved to be a successful strategy to overcome other types of EGFR-TKI resistance mechanism [32, 33].

There are some limitations of this study. Firstly, although all imaging studies were interpreted by two independent reviewers who were not aware of patients' molecular profiles, all clinical data were collected retrospectively. Secondly, only a small number of tissue samples were in adequate amounts for molecular analysis during the study period; therefore, unintentional selection bias might exist as it could not have been prevented. Thirdly, although the NGS-based cancer panel is a powerful tool for detecting hundreds of mutations, this screening platform cannot identify certain genetic alterations, such as gene amplification and rearrangement. There exist examples of these types of genomic variations, e.g., *MET* amplification and *ALK* rearrangement, which are associated with EGFR-TKI resistance [14, 34, 35]. Finally, we only included *EGFR* L858R-mutant tumors in this study. Patients with L858R and those with another prevalent EGFR-activating mutation, a small deletion in exon 19, have different EGFR-TKI treatment outcomes [36, 37]. It is conceivable that these two different activating *EGFR* mutations may not associate with the same mechanism of primary resistance to TKIs.

In conclusion, this study has identified a novel candidate for genetic predictor of primary EGFR-TKI resistance in *EGFR* L858R-positive lung adenocarcinomas. Patients with *EGFR* L858R-mutant lung adenocarcinoma have inferior EGFR-TKI treatment outcomes if they have a co-existing *MLH1* V384D variant. Future prospective clinical studies are warranted to confirm the prognostic importance of *MLH1* V384D, as well as to define appropriate combinations of anti-cancer therapeutics in treating tumors with concomitant existence of *EGFR* L858R and *MLH1* V384D alleles.

## METHODS

### Patients and study design

Patients were included if they had primary lung adenocarcinoma harboring the L858R mutation without a co-existing T790M mutation in *EGFR* and received their first-time EGFR-TKI treatment at Taipei Veterans General Hospital during the period from January 2009 to January 2013. Patients who had prior EGFR-TKI therapy or received EGFR-TKI in combination with other anti-cancer treatment were excluded. Patients who had adequate tumor specimens for further molecular testing were enrolled. This study was approved by the Institution Review Board of Taipei Veterans General Hospital.

If tumors progressed within 3 months of the initiation of EGFR-TKI therapy, we considered that the treatment was clinically ineffective and that these patients presented primary (or intrinsic) resistance. To discover candidate genetic variations that may associate with primary EGF-TKI resistance in *EGFR* mutant tumors, we performed genomic profiling of *EGFR* L858R tumors from 16 patients with long (> 1 year) progression-free survival (PFS) and 13 patients with short (< 3 months) PFS. NGS was performed to screen through a cancer-related gene mutation panel (Ion AmpliSeq Cancer Panel, Ion Torrent, Life Technologies); 739 mutation hotspot regions within 46 key cancer-related genes from the COSMIC database were examined. Distributions of genomic variants in the two groups of patients were compared. Genes with differential mutation status between two groups were further investigated in a total of 158 *EGFR* L858R tumors by PCR amplification and direct Sanger sequencing, and the association of candidate variants with differential tumor response to EGFR-TKIs was explored.

### Histopathology review and sample preparation

Consecutive tissue sections were prepared from each archived formalin-fixed paraffin-embedded (FFPE) pathology specimen and reviewed by pathologists; tumor areas were marked on deparaffinized unstained sections and manually dissected. Proteinase K-digested tissue extracts were subjected to genomic profiling tests. Genomic DNA was also prepared from available blood samples using the illustra blood genomicPrep Mini Spin Kit (GE Healthcare Life Sciences) according to the manufacturer's protocol.

### Next-generation sequencing

Genomic DNA from FFPE tumor tissues was quantified using the Qubit^®^ dsDNA HS Assay Kit and the Qubit^®^ fluorometer (Life Technologies); 10 nanograms were amplified by multiplex PCR using the Ion AmpliSeq Cancer Panel Primers Pool (Life Technologies). PCR amplicons were ligated with barcode adaptors using the Ion Xpress Barcode Adapters 1-16 Kit (Life Technologies), and subjected to emulsion PCR. Template was prepared by the automated Ion OneTouch System using the Ion OneTouch 200 Template Kit v2 DL, and DNA was sequenced on a 316 chip using the Ion PGM Sequencing Kit v2 and the Ion Torrent Personal Genome Machine (PGM, Ion Torrent, Life Technologies). Data were analyzed using the Torrent Suite software v3.0 and the Ion Torrent Variant Caller software v3.0. Variants were called when a minimum coverage of 500 reads was achieved and at least 5% of variant reads were identified.

### PCR and sanger sequencing

Exon 12 of the *MLH1* gene was amplified from genomic DNA by PCR using a forward primer (5′-CAGACTTTGCTACCAGGACTTGC-3′) and a reverse primer (5′-CTGCCTAGCCCTGCCACTAG-3′). PCR products were sequenced using the Sanger method. DNA sequences were analyzed by the Mutation Surveyor software (SoftGenetics, State College, PA).

### Statistical analysis

The objective tumor response was evaluated according to the revised RECIST criteria [34]. PFS was calculated from the date of starting EGFR-TKI therapy to the date of disease progression or death. The association between patient characteristics and *MLH1* mutation status was analyzed by chi-square and Fisher's exact tests. Kaplan-Meier survival curves were constructed and compared using the log-rank test. Cox regression models were built using a backward stepwise procedure for multivariate survival analysis. Analyses were carried out using PASW Statistics 18.0 (SPSS Inc., Chicago, IL).

## SUPPLEMENTARY MATERIAL AND FIGURE


