# Supplementary material for: MLH1 V384D polymorphism associates with poor response to EGFR tyrosine kinase inhibitors in patients with EGFR L858R-positive lung adenocarcinoma
Source: Oncotarget. 2015 Mar 10;6(10):8407–17. doi: 10.18632/oncotarget.3511 (PMC4480762; doi:10.18632/oncotarget.3511)
Supplement: Supplementary file 1 [file oncotarget-06-8407-s001.pdf]

MLH1 V384D polymorphism associates with poor response to EGFR tyrosine kinase inhibitors in patients with *EGFR* L858R-positive lung adenocarcinoma

Supplementary Material

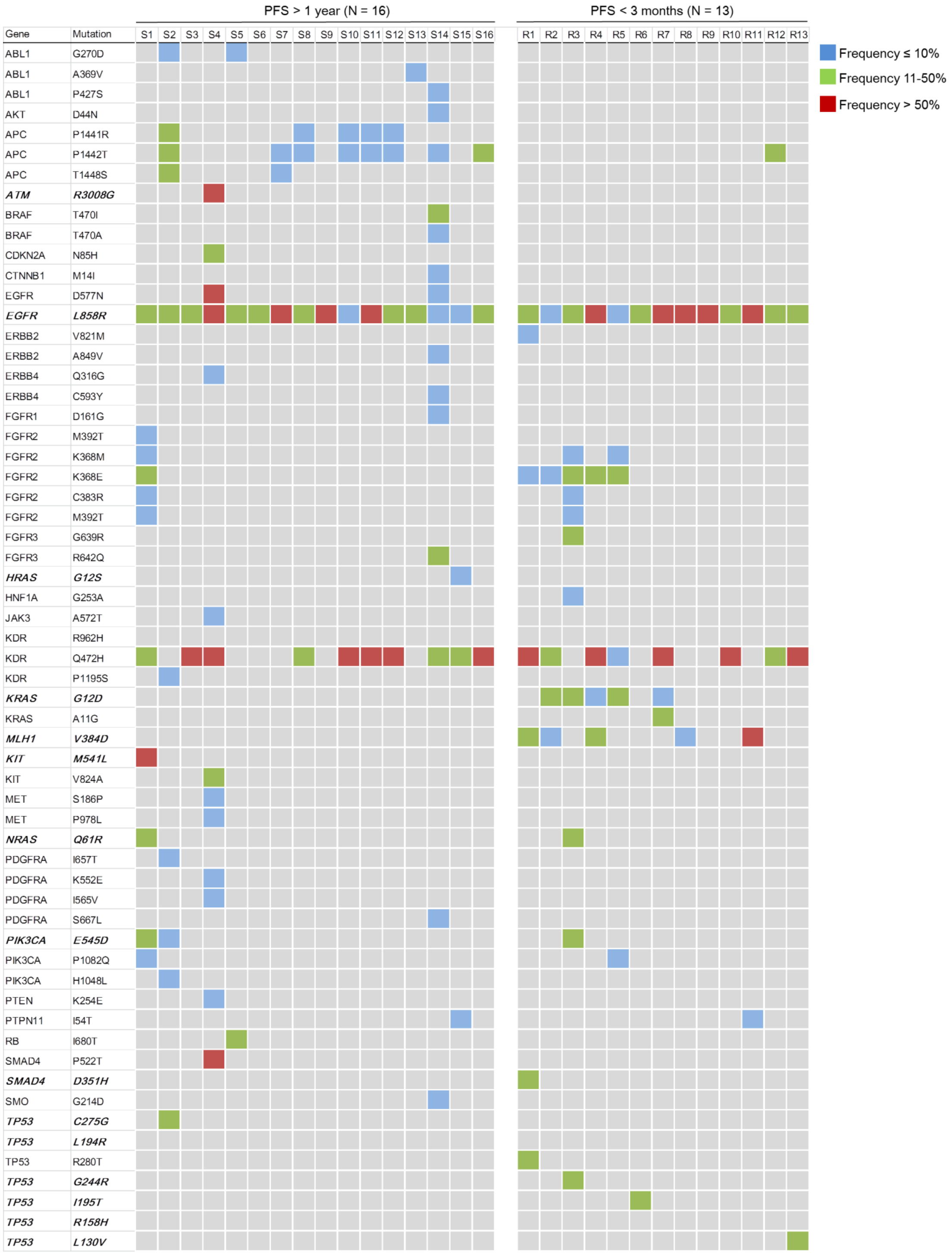

Supplementary Figure S1: Mutation profiles of *EGFR* L858R lung adenocarcinomas.

Amino acids variations within the hotspot regions of 46 cancer-related genes in individual *EGFR* L858R tumors are shown on the left in 2 groups, according to the progression-free survival (PFS) of patients. Frequencies of individual genetic variations detected by the IonTorrent software were grouped into three ranges and shown in different colors. Variations shown in bold and italic are hotspot mutations published in the COSMIC database.
